# REVERSING INSULIN RESISTANCE AND SARCOPENIA BY LOWERING TOXIC CERAMIDES IN OBESE NONHUMAN PRIMATES

**DOI:** 10.1093/geroni/igae098.0128

**Published:** 2024-12-31

**Authors:** Juan Palavicini, Andrew Murton

**Affiliations:** UT Health SA, San Antonio, Texas, United States; UTMB Health, Galveston, Texas, United States

## Abstract

Obese and old individuals are at heightened risk of developing insulin resistance, sarcopenia, and frailty. Specifically, we found that older obese men experience a diminished capacity to synthesize new muscle proteins in response to exogenous amino acids, a process pivotal for the maintenance of muscle mass and quality. Our ability to recreate this “anabolic resistance” to nutrition in lean young individuals through the acute infusion of lipid to induce obese-like ectopic lipid accumulation, suggests that the accumulation of lipid represents a pivotal mechanism by which the responsiveness of muscle to dietary amino acids diminishes with obesity. Ceramides are a class of lipids that have been associated with multiple negative metabolic effects and hallmarks of aging including insulin resistance, apoptosis, mitochondria disfunction, senescence, and inflammation. Using a variety of lipidomics approaches in combination with mouse models of obesity and diabetes, we have consistently found that ceramides accumulate in insulin sensitive organs, including muscle. Given prior evidence that protein synthesis is inhibited in human muscle cells subjected to ceramide treatment, which occurs in concert with the inhibition of mTOR signaling, a key driver of protein translation, we hypothesized that ceramides are major drivers of both insulin resistance and frailty. Here we will report how myriocin, an inhibitor of ceramide synthesis, restores insulin resistance in obese and diabetic mice. Findings from our ongoing pilot study in non-human primates to examine if myriocin improves insulin resistance and the anabolic response of muscle to diet in obese marmosets also will be reported.

